# P37 Molecular characterization of resistance and heteroresistance to cefiderocol in clinical Gram-negative isolates

**DOI:** 10.1093/jacamr/dlae136.041

**Published:** 2024-08-23

**Authors:** Francesca Young, Celeste Watson, Sylvia Rofael, Emmanuel Wey, Indran Balakrishnan

**Affiliations:** UCL Centre for Clinical Microbiology, University College London, London, UK; UCL Centre for Clinical Microbiology, University College London, London, UK; UCL Centre for Clinical Microbiology, University College London, London, UK; UCL Centre for Clinical Microbiology, University College London, London, UK; UCL Centre for Clinical Microbiology, University College London, London, UK

## Abstract

**Background:**

Treatment failure with cefiderocol has been attributed to heteroresistance, a phenomenon whereby clinically susceptible bacterial populations contain a minority resistant subpopulation.

**Objectives:**

To compare three antimicrobial susceptibility tests (AST) for detecting cefiderocol resistance/heteroresistance; to investigate the population dynamics of heteroresistant isolates in response to antibiotic exposure; and to characterize the molecular mechanisms underlying resistance and heteroresistance to cefiderocol.

**Methods:**

Forty-three Gram-negative clinical isolates were screened for cefiderocol susceptibility using disc diffusion, Etest and broth microdilution (BMD), according to EUCAST guidelines. Eleven isolates demonstrating heteroresistance were serially passaged in iron-depleted Mueller–Hinton II broth containing sub-inhibitory concentrations of cefiderocol (0.25xMIC) and then further passaged in the absence of antibiotic selection pressure. Changes in MIC and frequency of the heteroresistant phenotype were determined using Etests. Fourier transform infrared and MALDI-TOF spectroscopy were used to compare the molecular differences between resistant, heteroresistant and susceptible phenotypes.

**Results:**

Etest assays significantly underestimated cefiderocol resistance (*P*<0.001), with 32% categorical agreement, compared with 75% for disc diffusion, relative to the gold-standard BMD method. However, BMD is poor at detecting heteroresistance, identifying only 1/11 (9%), whilst Etests identified 3/11 (27%) (Figure 1). After passages under antibiotic selection pressure, 2/11 (18%) increased in MIC (mean: 3-fold). After subsequent antibiotic-free passages, 4/11 (36%) demonstrated increased MICs. Interestingly, this contrasted with isolates only passaged in antibiotic-free medium, in which 8/11 (72%) decreased in MIC, reverting to the more susceptible parental phenotypes. Molecular analyses highlighted characteristic differences between the spectra of parental populations and subpopulations, indicating complex mechanistic modifications in carbohydrate, lipid and protein complexes.

**Conclusions:**

Detection of clinically significant heteroresistance indicates that resistant subpopulations may exist amongst a phenotypically susceptible population, underscoring the need for heteroresistance detection methods in clinical settings. The heteroresistant phenotype was unstable and reverted to susceptibility unless exposed to cefiderocol. Interestingly, isolates exposed to antibiotic pressure often exhibit increases in MIC only after antibiotic removal. These findings suggest that cefiderocol exposure primes isolates for later development of resistance. Spectroscopic analyses confirm the presence of characteristic molecular fingerprints associated with cefiderocol susceptibility, which may inform our understanding of resistance.

**Figure 1.**
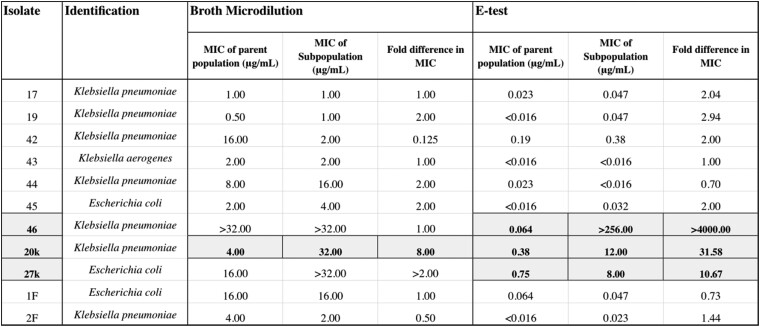
Detection of heteroresistance using BMD and Etest. Heteroresistance is identified where subpopulations exhibit ≥8-fold higher MICs than the parent population.

